# Integrating human–AI collaboration into translation education: A comprehensive protocol for assessment, diagnosis, and strategy development

**DOI:** 10.1371/journal.pone.0338089

**Published:** 2025-12-15

**Authors:** Xiaobin Ren, Ruoxuan Wang

**Affiliations:** 1 School of Foreign Languages, Guangxi University, China; 2 Asia-Pacific (Southeast Asia) Institute for Translation and Intercultural Studies, Guangxi University, China; Xi'an Jiaotong-Liverpool University, CHINA

## Abstract

In the era of artificial intelligence (AI), translation education faces pressing challenges to integrate human–machine collaboration into talent cultivation. This study protocol outlines a two-year mixed-methods project that focuses on developing, validating, and applying systematic tools for assessing human–machine collaborative competence among translation students and teaching competence among translation instructors. In the first stage, measurement instruments will be constructed and validated through thematic analysis, exploratory and confirmatory factor analysis, based on interviews, pilot testing, and large-scale surveys. The second stage will employ grounded theory, structural equation modeling, and regression analysis to identify the factors influencing students’ and teachers’ competence development. The third stage will investigate industry needs through semi-structured interviews with translation service professionals and descriptive statistical analysis across multiple domains. The fourth stage will assess classroom effectiveness via multimodal analysis of teaching and learning processes across five types of universities. Finally, a comprehensive training strategy will be designed, refined through action research pilot implementations, and validated by Delphi consultation with translation educators, industry specialists, and policymakers. By integrating empirical rigor with iterative validation, this study advances theoretical modeling of human–AI collaboration, establishes robust assessment tools for students and teachers, and delivers actionable training strategies that align translation education with evolving professional and industry needs.

## 1. Introduction

Recent research suggests that the integration of AI in higher education is reshaping teaching and learning in ways that offer both new opportunities and emerging challenges, influencing educators’ professional roles, emotional experiences, and instructional decision-making [1–3] as well as students’ engagement, learning autonomy, and cognitive processing in AI-mediated environments [4–6]. Against this broader backdrop of educational transformation, the rapid advancement of AI and large language models (LLMs) has also profoundly reshaped translation education [7,8]. Increasingly, translation classrooms are experimenting with AI-powered tools such as ChatGPT, DeepL, and Baidu Translate to support students’ learning processes [9–11]. Existing studies have explored how these tools can enhance language skills, including writing fluency [12], logical organization [13], oral communication [14], and error correction [15]. In the field of translation learning, scholars have advocated the integration of AI technologies into translator training [16] and proposed preliminary models of human–AI collaborative pedagogy [17]. At the same time, international research has generated valuable insights into interactive machine translation systems and their technical performance, highlighting improvements in translation quality and user interaction [18–20].

Despite these advances, several limitations remain. First, while AI-assisted translation is widely discussed, existing research rarely provides a systematic analysis or validated assessment of students’ human–AI collaborative competence. The absence of structured evaluation tools makes it difficult to measure learners’ abilities or identify their developmental gaps. Second, research on translation teachers has lagged far behind. Most studies have focused on teachers’ attitudes or acceptance of AI tools [e.g., 21, 22], while little attention has been paid to translation teachers’ human–AI collaborative teaching competence—a crucial factor influencing pedagogical effectiveness in AI-mediated environments. Third, although human–AI collaborative models for translation classrooms have been proposed [17,23], empirical evidence is limited. There is still limited robust multimodal evaluation framework capable of diagnosing teaching quality, interaction dynamics, and learning outcomes in real classroom contexts. Fourth, current literature seldom investigates industry-driven requirements for human–AI competence in professional translation practice. Without clear knowledge of industry expectations, translation training risks drifting away from workplace demands.

Against this backdrop, the present study seeks to fill these gaps by focusing on both students and teachers as dual subjects of inquiry. Specifically, it aims to (1) develop and validate assessment tools for translation students’ human–AI collaborative competence and teachers’ human–AI collaborative teaching competence, (2) analyze the mechanisms influencing these competencies through qualitative and quantitative methods, (3) investigate industry requirements for human–AI competence across translation tasks, (4) evaluate classroom practices via multimodal analysis, and (5) propose evidence-based strategies for cultivating human–AI collaborative competence in translator training. Through this comprehensive design, the study not only enriches the theoretical framework of translation education under intelligent conditions but also provides practical guidance for aligning higher education with the evolving needs of the translation industry.

## 2. Research procedure

### Ethics statement

This study was reviewed and approved by the Medical Ethics Committee of Guangxi University (Approval No.: GXU-2025–091). All research activities involving human participants, including interviews, classroom observations, and questionnaire surveys, will be implemented in accordance with the Ethical Review Methods for Biomedical Research Involving Human Subjects (Trial Version) and the Declaration of Helsinki. Prior to participation, all translation students, translation teachers, and industry professionals will be fully informed of the study’s purpose, procedures, potential risks, and their rights (including voluntary participation and the right to withdraw at any time). Written informed consent will be obtained from all participants before data collection begins. All personal identifiers will be removed during data processing, and data will be stored securely and be accessed only to the core research team.

This study follows a five-stage research procedure to integrate theory and practice. First, it develops and validates assessment tools for students’ human–AI collaborative competence and teachers’ teaching competence, establishing a solid foundation. Second, it analyzes influencing mechanisms using qualitative and quantitative methods. Third, it investigates industry requirements to align training with professional needs. Fourth, it employs multimodal analysis to evaluate classroom practices and identify improvement areas. Finally, it synthesizes findings to design and refine a comprehensive strategy for cultivating human–AI collaborative competence, ensuring coherence between educational theory, empirical validation, and practical application.

### Phase 1: Development of assessment tools for students and teachers

**(a) Development of the human–AI collaborative translation competence scale for students**

At the learner level, this study aims to construct a reliable and valid assessment tool to capture translation students’ competence in human–AI collaboration. Approximately 25 translation students will be invited to participate in semi-structured interviews between January and mid-February 2026 to identify key dimensions and competence indicators. Based on the thematic analysis, an initial scale will be developed by the end of February 2026 and piloted with around 150 students from March to April 2026. The item structure will undergo refinement through exploratory factor analysis (EFA) in April 2026. Subsequently, a larger sample of about 250 students will complete the revised scale during May 2026, and confirmatory factor analysis (CFA) will be used by the end of June 2026 to examine the validity and reliability. The final instrument, expected to be finalized in late June 2026, will provide a comprehensive measure of students’ abilities in areas such as technology use, collaborative interaction, critical evaluation, and learning strategies.

**(b) Development of the human–AI collaborative teaching competence scale for teachers**

At the teacher level, the study focuses on developing an assessment tool to evaluate translation teachers’ competence in human–AI collaborative pedagogy. Semi-structured in-depth interviews are planned with five expert teachers experienced in AI-assisted translation teaching between January and early February 2026, while classroom observations of approximately 20 frontline translation teachers’ classes will be carried out from mid-February to early March 2026 to capture key teaching practices. Thematic analysis will be used throughout February–March 2026 to construct initial dimensions and items. The draft scale will then be administered to approximately 120 teachers during April 2026, with EFA performed in late April to early May 2026 to refine the structure. Finally, the revised scale will be tested with about 250 teachers in May–June 2026, applying CFA by the end of June 2026 to establish its reliability and validity. The resulting tool, expected to be finalized in late June 2026, will systematically measure teachers’ competence in task design, technology integration, classroom management, and assessment practices in AI-mediated contexts.

### Phase 2: Analysis of influencing mechanisms

In this phase, the study adopts a constructivist epistemological stance, in which human interpretive meaning-making remains central and computational modeling is understood as a tool for clarification and verification. Guided by this perspective, the research follows an explanatory sequential mixed-methods logic that highlights the complementary relationship between qualitative interpretation and quantitative validation. The qualitative stage is used to uncover the experiential, cognitive, and pedagogical dimensions that shape human-AI collaboration, generating conceptually rich categories rather than merely producing variables. These categories inform hypothesis development and guide the structure of the subsequent quantitative analysis. Following quantitative testing, the study will return to the qualitative corpus to re-examine and contextualize the statistical pathways, ensuring that the theoretical model remains grounded in lived experience rather than being constrained by numerical abstraction.

**(a) Human–AI collaborative translation competence among students**

At the student level, the study will first conduct semi-structured, in-depth interviews with approximately 25 translation majors between July and early August 2026 to identify the critical factors influencing their human–AI collaborative translation competence. Using grounded theory, a theoretical model of influencing factors will be developed through August–September 2026. Based on this model, research hypotheses will be formulated in late September 2026 and subsequently tested with a larger sample of over 200 translation students. Structural equation modeling (SEM) and regression analysis will then be employed from mid-November to December 2026 to verify the proposed hypotheses, thereby clarifying the pathways through which individual characteristics, prior technological experience, and learning environments shape students’ competence in human–AI collaboration. Following the completion of statistical testing, the research team will return to the original qualitative corpus to compare and contextualize the quantitative pathways, ensuring that the explanatory model remains anchored in students’ lived collaborative practices rather than being reduced to numerical abstraction. The final analysis and reporting for this phase are expected to be completed by the end of December 2026.

**(b) Human–AI collaborative teaching competence among teachers**

At the teacher level, a similar approach will be applied. Semi-structured interviews are to be carried out with approximately 25 university translation teachers between July and early August 2026 to explore the key factors shaping their collaborative teaching competence in AI-mediated contexts. Grounded theory will guide the construction of a preliminary theoretical model through August–September 2026, from which hypotheses will be derived in late September 2026. These hypotheses will then be tested with data collected from about 200 translation teachers during October–November 2026, using SEM and regression analysis from mid-November to December 2026 to examine the relationships among variables. After the quantitative analysis, the study will return to the interview data to interpret how the identified pathways manifest in real instructional decision-making, thereby preserving the nuanced, situated nature of teachers’ human-AI collaborative pedagogy. The final analysis and reporting for this stage are expected to be completed by the end of December 2026, yielding an empirically validated framework that highlights the role of pedagogical design, technology integration, classroom management, and assessment practices in developing teachers’ competence for human–AI collaborative translation education.

### Phase 3: Industry needs assessment for human–AI collaborative competence

This phase aims to systematically investigate the translation industry’s requirements for human–AI collaborative competence, ensuring that translator training programs remain aligned with professional expectations and evolving market demands.

First, semi-structured in-depth interviews are scheduled with approximately 20 frontline experts from around 10 translation companies or language service providers between January and mid-February 2027, focusing on identifying industry perspectives and practical needs regarding technological application, post-editing skills, multimodal information processing, and ethical awareness.

Second, a large-scale survey will then be designed in late February 2027 and administered to professionals from March to mid-April 2027 across at least five specialized domains—such as medical translation, engineering translation, legal translation, financial translation, and scientific/technical translation—to capture both commonalities and divergences in required competencies.

Finally, qualitative data from interviews will undergo thematic analysis through March–April 2027, while quantitative survey data will be processed using descriptive statistics in May 2027. The integration of these findings will produce a detailed industry competence profile by June 2027, highlighting essential skill sets and domain-specific demands. This profile will provide a robust reference for aligning educational objectives, refining curricular content, and informing the design of evidence-based strategies for cultivating human–AI collaborative translation competence.

### Phase 4: Evaluation of human–AI collaborative teaching quality in translation classrooms

Following the development of measurement tools and the assessment of industry needs, the study will proceed to evaluate the quality and effectiveness of human–AI collaborative teaching practices in authentic classroom contexts. Sampling of five universities of different types—including comprehensive, science and engineering, foreign language, normal, and private institutions—will be finalized in early July 2027. From these institutions, at least 20 translation classes taught by frontline teachers will be observed between mid-July and late August 2027 as the primary units of analysis. During this period, classroom audio and video recordings, records of teachers’ instructional behaviors, learners’ process data, and human–AI interaction texts will be collected concurrently.

To ensure representativeness and ecological validity, Phase 4 will adopt purposeful sampling. Universities will be selected to reflect institutional diversity, and classes will be included only if they meet the following criteria: (a) the course is officially designated as a translation practice or translation technology course; (b) the instructor has integrated at least one AI-supported translation tool in classroom instruction; and (c) students have given voluntary consent to participate. Teachers and students who are unwilling to be recorded or whose classes involve confidential professional translation projects will be excluded from observation.

All classroom audio-video recordings, screen-capture logs, and human-AI interaction texts will be de-identified prior to analysis, with personal names and institutional identifiers replaced by coded labels. Data will be accessed only by the core research team. No data will be shared outside the project, and no classroom materials will be used for public dissemination or instructional evaluation. In accordance with the approval granted by the Medical Ethics Committee of Guangxi University (Approval No. GXU-2025–091), all participants will provide written informed consent, and data will be retained for five years before secure deletion.

Multimodal data cleaning and coding will take place from early September to early October 2027, after which this study will conduct multimodal analysis in October 2027 to examine dimensions such as goal attainment, quality of teacher–student interaction, effectiveness of AI tool integration, and students’ learning outcomes.

This stage aims to identify strengths and weaknesses of human-AI collaboration in real classroom settings and to reveal the degree of alignment—or mismatch—between classroom practices and industry requirements. A preliminary report summarizing diagnostic findings will be completed by late October 2027 to inform the subsequent phase of training strategy development.

### Phase 5: Strategy development for human–AI collaborative translator training

Building on the preceding phases, this stage focuses on formulating a systematic strategy for cultivating human–AI collaborative competence in translator education.

Preliminary strategies will be drafted in November 2027 based on findings from prior phases. Classroom-based pilot implementations will be conducted during November and December 2027 in translation courses across at least five types of institutions—comprehensive, foreign language, science and engineering, normal, and private universities. These pilots will test the strategies in authentic teaching environments, with iterative refinements made throughout November–December 2027 using action research and continuous feedback from teachers and students to ensure adaptability and responsiveness to practical challenges.

During January 2028, the Delphi method will be employed to conduct multiple rounds of consultation with a panel of 10 experts—comprising frontline translation educators, specialists from translation service companies, and policymakers—to validate and further refine the proposed framework.

By the end of January 2028, the finalized strategy will be consolidated, covering competence framework development, curriculum design, pedagogical innovation, and teacher training enhancement. By bridging technological and linguistic competencies and aligning classroom practices with industry expectations, this stage will deliver a systematic and transferable pathway for cultivating translation talent capable of thriving in AI-driven professional environments.

**Fig 1:**
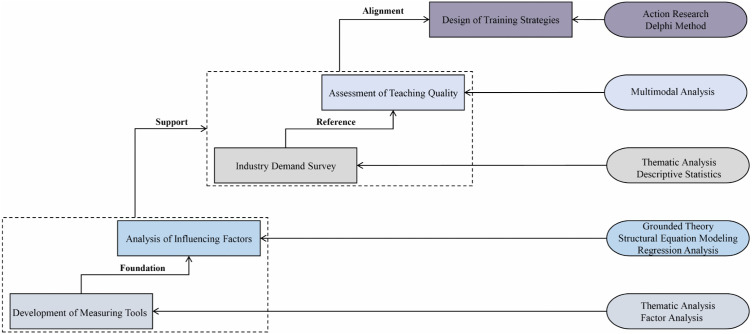
The figure illustrates the five sequential phases of the study: (1) development of assessment tools; (2) analysis of influencing mechanisms; (3) industry needs assessment; (4) classroom evaluation; and (5) strategy development. The arrows indicate the correspondence between each research phase and the specific methodological approaches employed on the right side, as well as the logical relationships among the phases. Dashed boxes group interrelated stages that are conceptually and procedurally connected, distinguishing them from other phases while highlighting their functional linkage within the overall research framework.

**Fig 1** demonstrates the five phases of the research procedure in this study.

## 3. Discussion

In terms of research focus, this study simultaneously addresses translation students’ human–AI collaborative competence and translation teachers’ human–AI collaborative teaching competence. Existing studies have primarily concentrated on the student population [e.g., 24–26], with comparatively limited attention to teachers. However, teachers’ competence in designing and implementing human–AI collaborative pedagogy plays a pivotal role in shaping learners’ skills and experiences [2,23,27] as it directly influences the effectiveness of talent cultivation. By incorporating both students and teachers as dual subjects of inquiry, this study advances a more comprehensive understanding of how collaborative competence is constructed and provides systematic evidence to support translation education reform.

Another distinctive contribution of this study lies in its emphasis on the translation industry’s expectations for human–AI collaborative competence—a perspective rarely addressed in existing research. While numerous studies have called for greater attention to learners’ competence cultivation [e.g., 28, 29], few have attempted to systematically align educational outcomes with actual market needs. Through industry surveys and expert interviews, this project identifies the profession’s core requirements and develops an evaluation framework for human–AI collaborative classrooms. Unlike prior studies that predominantly assess the technical performance of AI tools [e.g., 30], this framework is designed to evaluate educational effectiveness, thereby enhancing the efficiency and relevance of translator training.

Methodologically, this study places particular emphasis on the use of action research. After developing the preliminary training strategy for human–AI collaborative competence, the framework will undergo multiple rounds of adjustment and refinement across pilot institutions. Such iterative improvement through classroom-based practice is rarely adopted in current translator training research, which has largely relied on cross-sectional designs and seldom engaged in systematic follow-up or modification of proposed teaching recommendations [e.g., 31, 32]. In addition, the study incorporates the Delphi method to conduct expert-based validation of the final training strategy. This combination not only ensures the feasibility and scientific rigor of the proposed framework but also enhances its credibility and transferability in diverse educational and professional contexts.
